# Beyond late gadolinium enhancement: the key role of diffuse myocardial fibrosis in severe aortic stenosis - an Equilibrium Contrast CMR study

**DOI:** 10.1186/1532-429X-13-S1-O39

**Published:** 2011-02-02

**Authors:** Andrew S Flett, Dan M Sado, Giovanni Quarta, Olivier Huttin, Derek Hausenloy, Sanjay M Banypersad, David Lawrence, Denis Pellerin, Christopher Mcgregor, Andrew M Taylor, James C Moon

**Affiliations:** 1 The Heart Hospital, London, UK; 2 Great Ormond Street Hospital for Children, London, UK

## Background

In severe aortic stenosis (AS), hemodynamics and conventional indices do not fully explain symptoms, prognosis or treatment response. We hypothesize that diffuse myocardial fibrosis (DMF) is a key missing factor in AS. This can now be accurately measured non-invasively using equilibrium contrast CMR (EQ-CMR) [1] involving a primed gadolinium infusion, T1 measurement pre- and post-infusion, and direct measure of blood volume of distribution (1-hematocrit). The derived myocardial volume of distribution (Vd_(m)_) correlates strongly with histological diffuse myocardial fibrosisin AS and this calibration can convert Vd_(m)_ to DMF%. Cell volume can be calculated as 1-DMF%*LV mass.

## Methods

63 severe AS patients with planned valve replacement underwent baseline and follow up EQ-CMR. Twenty normal controls were included. Baseline and follow-up assessment included NYHA, ECG, echocardiography (for diastolic function and valve area/velocities), BNP and six minute walk test (6MWT). Follow up was at 6-months (2 declined, 4 late deaths, 13 pacemakers, 11 outstanding, leaving 33). EQ-CMR results were expressed as Vd_(m)_/DMF% (continuous variable or severity tertiles), or cell volume.

## Results

### Baseline

**Figure 1:**
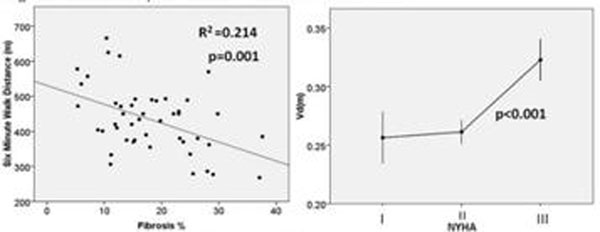
Relationship of DMF with 6MWT and NYHA class.

AS patients had more fibrosis than controls (Vd_(m):_0.27±0.04 vs 0.24±0.04; DMF:17% vs 11%, p = 0.003) with a wide range (Vd_(m)_:0.20-0.39; DMF:4-42%). Breathless patients had more DMF (NYHA class III/IV vs I/II: Vd_(m)_:0.32±0.03 vs 0.26±0.04; DMF:15% vs 27%, p<0.001). DMF correlated with 6MWT (inversely, figure 1, r^2^=0.22, p=0.001) and aortic valve area (r^2^=0.21, p=0.001). DMF only correlated with EF in patients with LV impairment (n=15, r^2^=0.47, p=0.01). Severe DMF patients had worse diastolic function (p=0.029). In a multivariate analysis of all parameters classically associated with 6MWT distance, the only independent predictor was DMF (p=0.04). On univariate analysis there was a weak correlation with BNP and age.

### Follow up

**Figure 2:**
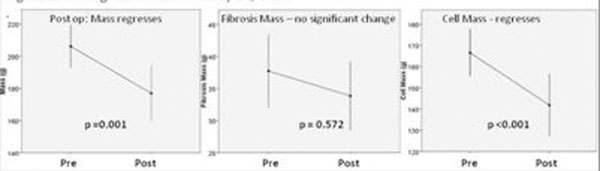
LVH regression redefined by EQ-CMR.

Overall, patients improved at follow-up (6MWT, EF, BNP, LV mass, LV volumes). However, only patients with severe fibrosis improved their exercise capacity (p=0.03). LVH regression (202g vs 183g, p=0.002) was shown to be cellular (161g vs. 142g, p<0.001) rather than fibrosis (36g vs 34g p=0.572) resolution, figure 2.

## Conclusion

In this first clinical EQ-CMR study of severe AS, DMF is higher when there is LV impairment, diastolic dysfunction and more severe stenosis. DMF is the single best predictor of pre-op exercise capacity and post-op improvement. EQ-CMR shows that at 6-month post valve replacement LVH regression is predominantly reduced cell rather than fibrosis volume. EQ-CMR for the non-invasive measurement of DMF appears to be a significant cardiological advance.
